# Secreted venom allergen-like proteins of helminths: Conserved modulators of host responses in animals and plants

**DOI:** 10.1371/journal.ppat.1007300

**Published:** 2018-10-18

**Authors:** Ruud H. P. Wilbers, Roger Schneiter, Martijn H. M. Holterman, Claire Drurey, Geert Smant, Oluwatoyin A. Asojo, Rick M. Maizels, Jose L. Lozano-Torres

**Affiliations:** 1 Laboratory of Nematology, Plant Sciences Group, Wageningen University and Research, Wageningen, The Netherlands; 2 Division of Biochemistry, Department of Biology, University of Fribourg, Fribourg, Switzerland; 3 Wellcome Centre for Molecular Parasitology, Institute of Infection, Immunity and Inflammation, University of Glasgow, United Kingdom; 4 Department of Chemistry and Biochemistry, Hampton University, Hampton, Virginia, United States of America; University of Pennsylvania, UNITED STATES

## Abstract

Despite causing considerable damage to host tissue at the onset of parasitism, invasive helminths establish remarkably persistent infections in both animals and plants. Secretions released by these obligate parasites during host invasion are thought to be crucial for their persistence in infection. Helminth secretions are complex mixtures of molecules, most of which have unknown molecular targets and functions in host cells or tissues. Although the habitats of animal- and plant-parasitic helminths are very distinct, their secretions share the presence of a structurally conserved group of proteins called venom allergen-like proteins (VALs). Helminths abundantly secrete VALs during several stages of parasitism while inflicting extensive damage to host tissue. The tight association between the secretion of VALs and the onset of parasitism has triggered a particular interest in this group of proteins, as improved knowledge on their biological functions may assist in designing novel protection strategies against parasites in humans, livestock, and important food crops.

## Introduction

Upon infection, helminth parasites establish an intricate relationship with their host. Helminths cause considerable damage during host invasion, migration through host tissues, and feeding on host cells [1, 2], but infections by these parasites can nonetheless be very persistent and last for several decades. Helminths are masters in manipulating host defense responses [1, 3], thereby creating a suitable environment for their survival and simultaneously limiting excessive damage due to host immune responses.

Excretory/secretory (ES) products are regarded as the tools employed by helminth parasites to control host defense responses. Recently, it was shown that ES products of helminth parasites reflect their diversity in lifestyles and hosts and therefore have little in common between plant and animal parasites [4]. However, members of the alternatively named Sperm-coating protein/Tpx/antigen 5/pathogenesis-related-1/Sc7 (SCP/TAPS) or cysteine-rich secretory proteins/antigen 5/pathogenesis-related 1 (CAP) protein superfamily are ubiquitously present in ES products of helminth species that parasitize plants and animals. Although a uniform nomenclature was proposed previously [5], helminth CAP proteins still go by different names, including activation-associated secreted proteins (ASPs) or most commonly used venom allergen-like proteins (VALs or VAPs).

The expression of VALs is specifically up-regulated during parasitic phases of the life cycle of helminths, which could point to a role in host–parasite interactions [6–9]. The presence of VALs in secretions of both plant- and animal-parasitic helminths suggests that these proteins are important for the establishment of persistent infections in both plants and animals. It is possible that conserved structural properties in VALs provide a diverse group of parasites a robust platform for modulating host responses in both plant and animal kingdoms. However, a question that remains unanswered is whether VALs from plant and animal parasites could have conserved functions based on common biochemical properties of these secreted proteins.

Recent reports have shed new light on structural properties, biochemical modes of action, and functions of secreted VALs of parasitic helminths. However, most of these reports have been published in specialized journals dedicated to either medical and veterinary biology or plant pathology. Here, we present an interdisciplinary review of the latest findings from phylogenetic analyses, x-ray crystallography, and functional studies on VALs from parasitic helminths. The aim of this review is to explore conserved mechanisms underlying the role of VALs as modulators of host responses during parasitism with an emphasis on their potential impact on common concepts in plant pathology and human and/or animal parasitology.

### Phylogenetic analysis of nematode VALs reveals no clear links with parasitism

**Fig 1:**
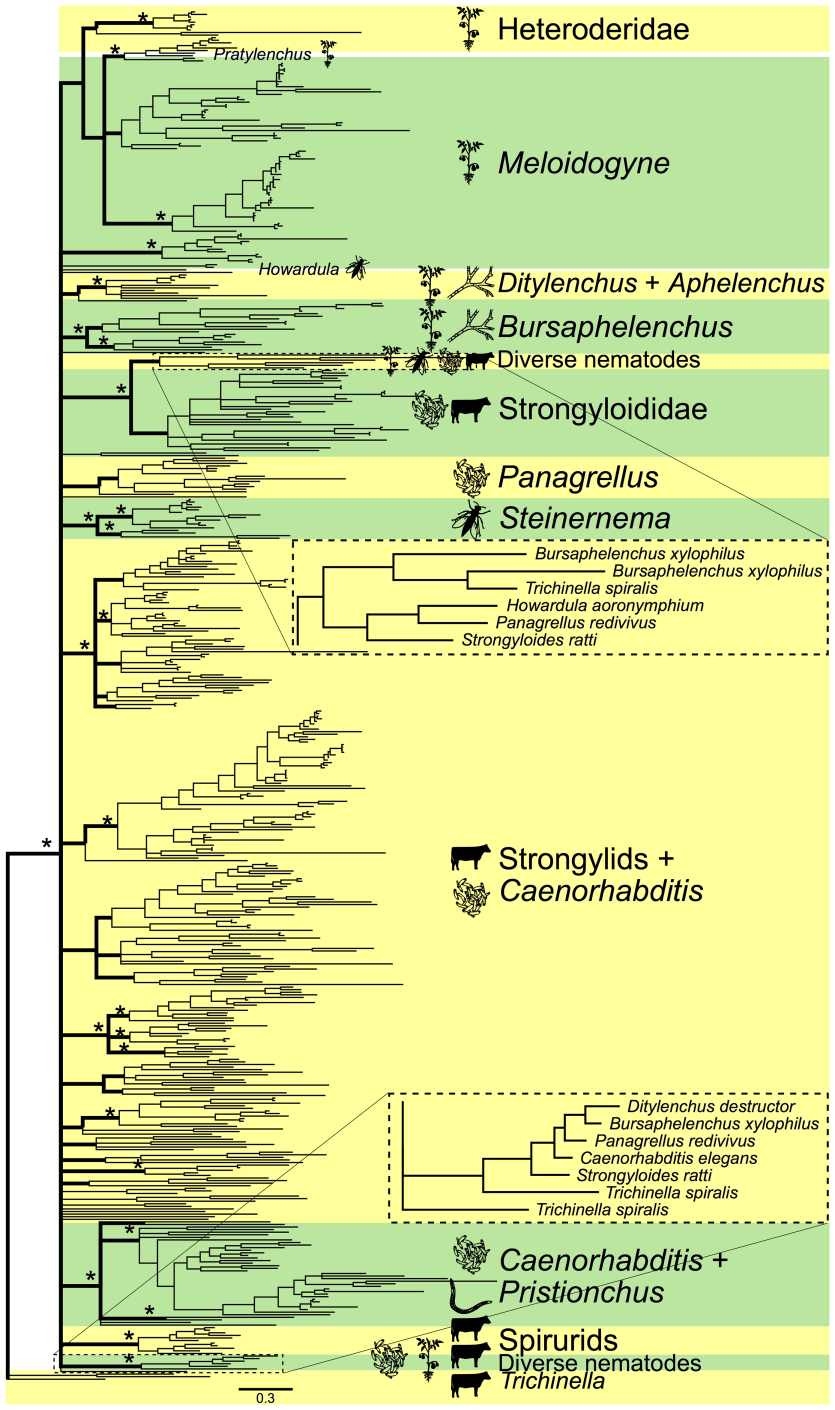
Bayesian tree of VALs in nematodes. Asterisks indicate main branches (bold) well-supported by the Bayesian or maximum likelihood analysis. Icons represent feeding types (plant parasitism, fungal feeding, bacterial feeding, nematode predation, insect parasitism, and vertebrate parasitism). Figure insets reveal two clusters with nematode species of plant- and animal-parasitic and free-living species. Taxon names, support values, and methods for construction can be found in S1 Fig. VAL, venom allergen-like protein.

Multiple lineages within the nematode phylum have independently evolved the ability to parasitize plants or animals. Modelling the evolutionary history of nematode VALs, based on overall sequence diversity, may reveal links between patterns of diversification within this protein family and particular lifestyles or host organisms. To this end, we generated a Bayesian tree of available VAL sequences from plant- and animal-parasitic nematodes as well as their free-living close relatives from the distal end of the nematode tree (Fig 1 and S1 Fig, S1 Table) [10]. First of all, we observed no separate clade of VALs uniquely associated with parasitism among nematodes. So, based on overall sequence diversity in VALs, we found no clear distinction between VALs from parasitic nematodes and free-living nematodes. Similarly, we also found no evidence for a clear separation between VALs from different lineages of plant parasites and VALs from different lineages of animal parasites, which could have pointed at specific adaptations to living on either host plants or animals. Instead, several VALs from plant-parasitic nematodes (*Ditylenchus destructor* and *Bursaphelenchus xylophilus*), animal-parasitic nematodes (*Howardula aoronymphium*, *Strongyloides ratti*, and *Trichinella spiralis*), and free-living nematodes (*Panagrellus redivivus*) clustered together (Fig 1 insets). Moreover, VALs from related parasitic and free-living nematode species clustered into separate clades, which points at functional diversification in a common ancestor of these parasitic and free-living lineages. By contrast, some clusters contain large numbers of homologous VALs from the same nematode genus, which indicates extensive recent functional diversification. The large difference in numbers of VALs present in nematode genomes is striking, ranging from a few (*H*. *aoronymphium*, *Brugia malayi*) to over a hundred (*Ancylostoma caninum*). Gene family expansion also does not seem to be linked to a particular lifestyle or host organism. Altogether, our phylogenetic analysis revealed no clear link between overall sequence diversity in VAL genes in nematodes and parasitism in plant and animals.

### Helminth VALs bind lipids and other hydrophobic structures

Helminth VALs are classified as SCP/TAPS proteins, which include a range of structurally related proteins found in a wide range of eukaryotes [5]. Ample structural information exists on eukaryotic SCP/TAPS proteins, but so far only a handful of structures for helminth VALs have been resolved. Similar to eukaryotic SCP/TAPS proteins, the core of helminth VALs is a structurally conserved approximately 15 kDa cysteine-rich CAP domain (Pfam00188), which typically has limited sequence identity [11–26]. SCP/TAPS proteins are generally made up of single CAP domains, which are sometimes covalently linked to other functional domains [20, 27]. A higher degree of structural complexity is seen in helminth VALs as they can comprise a single CAP domain with the ability to form homodimers (like *Oo*-ASP-1 from *Ostertagia ostertagi* [15]), two covalently linked CAP domains (like *Na*-ASP-1 from *Necator americanus* [28]), or even four covalently linked CAP domains (*P*. *redivivus* gene Pan_g9869.t1, genome PRJNA186477 WormBase ParaSite). In addition, helminth VALs can be found fused to other protein family sequences such as the ShK toxin domains (Pfam01549) in *Toxocara canis* [29].

**Fig 2:**
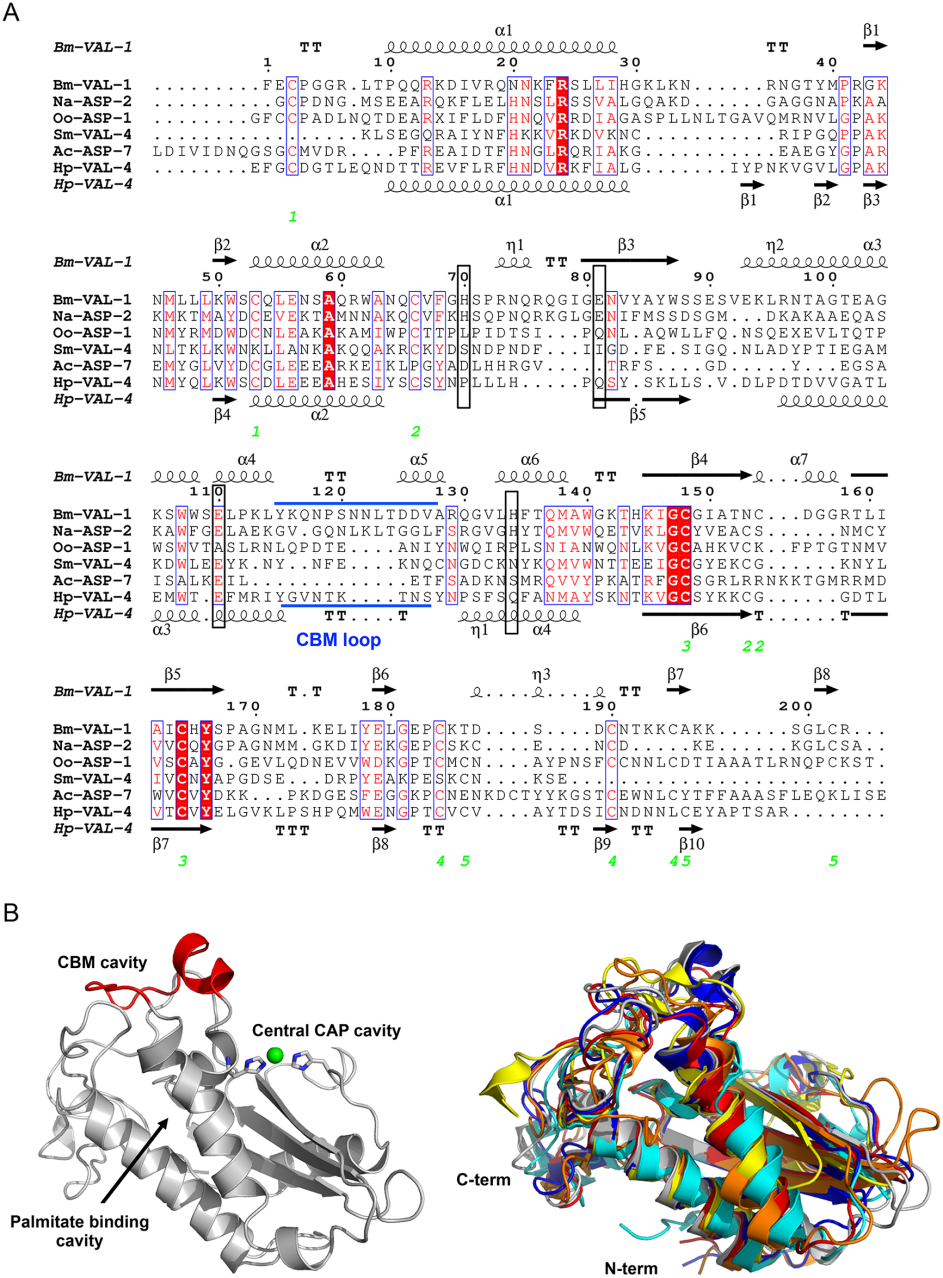
Comparison of helminth VALs. (A) The sequences of helminth VALs with known structure (see below) are aligned with clustalW2, and the secondary structural features are illustrated with the coordinates of *Bm*-VAL-1 and *Hp*-VAL-4 using ESPript. The different secondary structural elements shown are alpha helices as large squiggles labeled (α), 3_10_-helices as small squiggles labeled (η), beta strands as arrows (β), and beta turns (TT). Identical residues are highlighted in solid red, and conserved residues are shown in red. The locations of the cysteine residues involved in disulfide bonds are numbered in green. The location of the CBM loop is shown in blue. The position of amino acid residues constituting the CAP tetrad are marked with a black box. (B) *Bm*-VAL-1 is given as a representative structure in which the different binding cavities are indicated. The CBM loop is depicted in red, and coordination of the divalent cation Zn^2+^ is shown in green. Superimposed structures of helminths VALs with a single CAP domain are given to illustrate structural differences among the different family members. The represented structures are *Na*-ASP-2 in blue [13], *Oo*-ASP-1 in orange [15], *Ac*-ASP-7 in cyan [90], *Sm*-VAL-4 in yellow [44], *Hp*-VAL-4 in red [12], and *Bm*-VAL-1 in gray [16]. ASP, activation-associated secreted protein; CAP, cysteine-rich secretory proteins/antigen 5/pathogenesis-related 1; CBM, caveolin-binding motif; VAL, venom allergen-like proteins.

The CAP domain itself adopts a characteristic alpha-beta-alpha sandwich fold with flexible loop regions and is further stabilized by disulfide bonds [11, 13, 14]. The lengths of the flexible loops, β-strands, and α-helices vary between different proteins, making it difficult to accurately model the structures of these proteins. An alignment of the amino acid sequences from single CAP domain VALs of helminths, for which the structures have been resolved, illustrates that conservation among these sequences is mainly found in structural properties, like disulfide bonds, α-helices, and β-strands (Fig 2A). Superimposing VAL structures on top of each other nicely illustrates that loop regions in between these secondary structures as well as N- and C-termini are very distinct (Fig 2B). Importantly, these loop regions constitute up to 50% of the overall VAL structure.

All reported SCP/TAPS protein structures have a large central CAP cavity, which are either cysteine-rich secretory protein (CRISP)-like or non-CRISP-like. The CRISP-like CAP cavity is characterized by a tetrad of residues, consisting of two histidine and two glutamic acid residues that bind divalent cations including Zn^2+^ and Mg^2+^ [14, 20, 30–38]. This tetrad was shown to be important for Zn^2+^ binding and the heparan-sulfate dependent inflammatory modulation mechanisms of the cobra CRISP protein natrin [38]. However, the majority of available sequences of plant and animal parasite VALs are suggestive of non-CRISP-like protein structures because they lack the histidine residues of the CAP tetrad [12]. Furthermore, our recent analysis of the *Bm*-VAL-1 structure reveals that the central CAP cavity is connected to other cavities by channels that can serve as pathways for water molecules, cations, and small molecules [16].

Given the shared structural topology of the CAP domain, it seems likely that CAP proteins exert a fundamentally similar biochemical function within their respective environments [20, 39, 40]. However, the exact nature of this shared biochemical function among CAP proteins remains poorly defined. Recent evidence shows that distinct pockets in the CAP domain bind lipids, such as leukotrienes, sterols, and negatively charged phospholipids [25, 41, 42]. CAP proteins from yeast (pathogen-related yeast protein [Pry1] and Pry2) bind sterols and fatty acids at two distinct lipid-binding sites [42, 43]. These two well-defined lipid-binding pockets are a flexible loop that binds sterols (called the caveolin-binding motif [CBM] loop) and a hydrophobic channel, which is formed by two parallel α-helices, the so-called palmitate-binding cavity. Furthermore, it was shown that the palmitate-binding cavity of tablysin-15 from the saliva of the horsefly *Tabanus yao* binds leukotrienes and thereby inhibits their pro-inflammatory effects [25].

Lipid-binding properties have recently also been demonstrated for helminth VALs using mutant yeast cells that lack their endogenous CAP proteins Pry1 and Pry2 and are deficient in sterol export. Expression of *Na*-ASP-2, *Sm*-VAL-4, *Hp*-VAL-4, or *Bm*-VAL-1 in these mutant cells restores their ability to export sterol [12, 16, 44]. These results also indicate that the CAP tetrad residues are not required for sterol export as both *Sm*-VAL-4 and *Hp*-VAL-4 lack the tetrad histidine residues. Sterol binding by *Bm*-VAL-1, on the other hand, can be inhibited with EDTA and therefore seems to be dependent on the binding of divalent cations. Besides sterol binding, BmVAL-1 is also able to bind palmitate in vitro with comparable affinity as tablysin-15 [16]. The two parallel α-helices that constitute the palmitate-binding cavity are structurally well conserved among other helminth VALs (Fig 2B), which suggests that these VALs likely bind palmitate or similar hydrophobic ligands as well. These studies introduce VALs as a new protein family of parasitic helminths with lipid-binding properties, just like nematode polyprotein antigens and/or allergens (NPAs), nematode fatty acid-binding proteins (nemFABPs), and fatty acid- and retinol-binding proteins (FARs) [45].

Altogether, helminth VALs share a conserved alpha-beta-alpha sandwich structure that is typical for CAP proteins. However, the length of different α-helices and β-strands and the composition of loop regions determine approximately 50% of their overall structure. This supports the idea that the CAP domain may act as a stable but versatile molecular scaffold for diversification of the entire VAL gene family. Also, evidence indicates that this stable CAP domain allows helminth VALs to sequester small hydrophobic ligands [12, 16]. This could be a potential mechanism used by VALs to modulate immune responses in their host.

### Biological functions of VALs from plant parasites

Plant-parasitic nematodes use an oral stylet to perforate plant cell walls, deliver secretions into cells, and extract low molecular weight compounds from living plant cells. VALs have attracted attention because they are among the very few molecules in stylet secretions that seem to be important for persistent nematode infections in plants and animals [46]. Following the discovery of *Mi*-VAP-1 from the root-knot nematode *Meloidogyne incognita* in 2000 [17, 47], research has focussed on the role of VALs as activators and suppressors of host immune responses. For instance, while studying the function of *Gr*-VAP-1 from the potato cyst nematode *Globodera rostochiensis*, it was found that binding of this protein to the extracellular cysteine protease Rcr3^pim^ in tomato triggers a defense-related hypersensitive response mediated by the surface-localized immune receptor Cf-2 [8]. Interestingly, tomato genotypes carrying the Rcr3^pim^ protein but lacking the matching immune receptor Cf-2 were more susceptible to nematode infections than plants lacking both Rc3^pim^ and Cf-2, suggesting that *Gr*-VAP-1 interacts with Rcr3^pim^ to suppress host immunity.

The expression of VAL genes in plant-parasitic nematodes is regulated in association with host invasion and subsequent migration through host tissues [8, 19, 48–54]. Proteomic analyses of chemically induced secretions from infective juveniles showed the presence of VALs along with different classes of plant cell wall-degrading enzymes [8]. Plant-parasitic nematodes utilize a large repertoire of plant cell wall-degrading enzymes to break down the protective layer of cellulose and other carbohydrate polymers around cells during migration. A knockdown of stylet-secreted cell wall-degrading enzymes by RNA interference in infective juvenile nematodes can halt host invasion [55]. Similarly, a knockdown of VAL expression in migratory plant-parasitic nematodes can also reduce their ability to migrate inside host plants and successfully establish a permanent feeding site [50, 56, 57]. These findings suggest that stylet-secreted VALs are important for modulating host responses, particularly during the migration of the nematodes through host tissues and the early stages of an infection.

Ectopic expression of nematode VALs (*Gr*-VAP-1, and *Hs*-VAP-1 and *Hs*-VAP-2 from *Heterodera schachtii*) in the extracellular matrix of transgenic plants significantly increases their susceptibility to plant-parasitic nematodes [57]. Interestingly, these plants also proved to be more susceptible to infections by fungi, bacteria, and oomycetes, all of which have entirely different infection strategies [57]. Furthermore, ectopic expression of nematode VALs suppresses the growth inhibition response that normally occurs when young plants are constantly exposed to the flagellin peptide flg22. Flg22 is recognized as a pathogen-associated molecular pattern by surface-localized immune receptors in plants [58, 59]. Whole transcriptome analysis of plants ectopically expressing nematode VALs suggests that enhanced susceptibility involves plant cell wall modifications, lipid signalling, and extracellular protein processing [57]. In a different experimental setup, ectopic nematode VALs suppress a defense-related hypersensitive response in plant cells mediated by surface-localized, but not by cytoplasmic, immune receptors. Taken together, stylet-secreted VALs of plant-parasitic nematodes most likely enhance overall susceptibility of host plants to nematode infections by suppressing plant innate immunity mediated by surface-localized receptors.

### Biological functions of VALs from animal parasites

Initially, the major focus of study for animal parasite VALs was their immunogenic properties. Following the discovery in the 1990s that dominant secreted proteins of hookworm larvae are the VAL family members ASP-1 and ASP-2 [22, 28], they were considered ideal candidates for vaccine development. Subsequently, VALs from a wide range of animal and human parasites have shown substantial degrees of protective immunity as vaccines [60–65]. *Na*-ASP-2 was explored as a potential human hookworm vaccine candidate, but despite successes in animal experiments [66–68], vaccine development was halted due to allergic reactions in previously exposed individuals in a clinical trial [69]. Although adverse, this outcome emphasized the immunogenicity of the VALs in natural helminth infection.

The ubiquitious presence and frequent dominance of VALs in helminth ES products—and their up-regulation during parasitic phases of the life cycle—point to a major role in host–parasite interactions. VALs are among the most abundant secreted proteins in the intestinal cattle parasites *Cooperia oncophora* and *O*. *ostertagi*, where they are prevalently upregulated in parasitic stages [70], and in the rodent model nematodes *Heligmosomoides polygyrus* [6] and *Nippostrongylus brasiliensis* [9]. A fascinating example is presented by *Strongyloides* nematodes, which can follow either a free-living life cycle or a parasitic cycle through mammals; multiple VAL family members show preferential expression in the parasitic adult worm compared to the free-living form of the same species [7]. Furthermore, predicted VALs from the trematode *Schistosoma mansoni* are up-regulated during parasite infective stages, indicating a role in invasion of the human host [71]. *Sm*-VAL-4 has been detected in human skin following invasion of *S*. *mansoni* cercariae [72]. In the filarial nematode *B*. *malayi*, *Bm*-VAL-1 is highly expressed in the mosquito-borne L3 stage prior to entry into the mammalian host, and is re-expressed subsequently by later stages [73]. ASP/VAL genes of *A*. *caninum* and *T*. *canis*, the dog hookworm and roundworm respectively, are abundantly expressed in invasive and migratory larval stages [74, 75], as are VALs in the adult stages of *A*. *caninum*, *Haemonchus contortus*, and *N*. *americanus* [76–78]. Environmental niche is also linked to VAL expression, which is significantly higher in mucosal-dwelling larvae of *Teladorsagia circumcincta* compared to larvae from the lumen [79]. VALs therefore seem to be expressed at stages of the parasite life cycle where maximal contact occurs between parasite and host, whether this is transmission, tissue migration, or feeding.

Location of expression in the parasite also hints at function, with many helminth VALs being expressed in secretory glands. Staining for the glycan found on *H*. *polygyrus* VAL-1 and VAL-2 identified a series of structures on the exterior cuticle in contact with host tissues [6]. Several *A*. *caninum* ASP proteins localize to the pharyngeal glands and glandular esophagus, which produce hookworm ES secretions [26, 80]. *S*. *mansoni* VALs also localize to the esophageal gland [81]. *Ov*-ASP-1, an ASP/VAL transcript from the human parasite *Onchocerca volvulus*—causal agent of river blindness—is localized in the glandular esophagus of L2 and L3 larvae and secreted via degranulation following the invasion of the host [63].

Though both timing and location of expression of VALs point to roles in parasite–host interactions, relatively few of these proteins have a well-defined physiological function. *Na*-ASP-2 binds to human B cells via CD79A, triggering downregulation of receptor signaling pathways [82]. *Na*-ASP-2 also induces neutrophil migration and accumulation within tissues, a potentially pro-inflammatory activity that may aid tissue migrating larvae through increased tissue permeability [83]. This contrasts with other VALs that suppress immune responses once a parasite is tissue-dwelling. For example, neutrophil inhibitor factor (NIF) from *A*. *caninum* binds to neutrophils via the integrin CD11b/CD18 and blocks their adhesion to vascular endothelial cells and oxidative bursts [84–86]. Hookworm platelet inhibitor (HPI) is secreted from adult *A*. *caninum* at the site of intestinal attachment, where it inhibits platelet aggregation and adhesion, allowing continuous feeding without blood clotting [87, 88]. *Sm*-VAL-9 from *S*. *mansoni* induces differential expression of matrix metalloproteinases in macrophages and modulates host extracellular matrix remodeling gene expression in both its vertebrate and snail hosts [89].

Importantly, care should be taken when results obtained from in vitro studies with recombinant helminth proteins are extrapolated to the in vivo situation. First of all, these proteins are often studied at seemingly supraphysiological concentrations. Furthermore, differences in protein folding and post-translational modifications (e.g., N-glycosylation) between native and recombinant helminth proteins could significantly influence binding to target cells or biological activity.

Taken together, the pattern of animal-parasitic VAL evolution and expression suggests that most family members fulfill core functions at the host–parasite interface, with rapid diversification and adaptation to each host species.

### Concluding paragraph

The expression pattern of VALs during invasion and migration of host tissues by both plant- and animal-parasitic helminths suggests that these proteins might have conserved mechanisms of immune modulation in their respective host. However, sequence analysis suggests an independent evolution of these functions. In recent years, several VAL family members have been functionally characterized (summarized in S2 Table) and revealed modulation of similar biological processes in plant and animal hosts, like suppression of innate immune responses and remodeling of the extracellular matrix. Furthermore, VALs reveal a conserved CAP domain that allows them to sequester small hydrophobic ligands (e.g., cholesterol and palmitate), but little is known about endogenous ligands that are bound during parasitism. Therefore, future research should focus on the specificity for different lipid ligands and how these ligands can be involved in the immunomodulatory properties of helminth VALs.

## Supporting information

S1 Fig A Bayesian phylogenetic tree of the functional domains of VAL genes in nematodes. For multidomain proteins, each domain was included separately. *Trichinella spiralis* served as an outgroup. The alignment was created in BioEdit version 7.2.5 using ClustalW 1,4 and was further manually refined. The Bayesian tree was created using MrBayes version 3.2.6 and run for 10 million generations with 4 chains in 4 parallel runs using a mixed amino acid substitution model. Runs converged after a burnin of 2 million generations and used the WAG substitution model. Posterior probabilities are given above the branches. Displayed below the branches are the bootstrap percentages of a fast maximum likelihood tree run on the same dataset with RAxML version 8.2.10 using the WAG substitution model with 1000 bootstraps. All sequences that were used to constrict this tree are listed in S1 Table. VAL, venom allergen-like protein; WAG, Whelan and Goldman.
(PDF)

S1 Table Sequences used in the creation of the phylogenetic trees. Sequences were gathered by blasting published genomes, EST databases and GenBank with VAL sequences or taken from literature references. Genomes were blasted on Wormbase Parasite (http://parasite.wormbase.org) with the exception of *B*. *xylophilus* (GeneDB: http://www.genedb.org), *S*. *ratti* (https://www.sanger.ac.uk/cgi-bin/blast/submitblast/strongyloides), *P*. *coffeae* (https://www.ncbi.nlm.nih.gov), and *H*. *aoronymphium* (http://nematodes.org/downloads/959nematodegenomes/blast/db/Howardula_aoronymphium_clc_1.fna). EST databases were blasted on Nematode.net (http://nematode.net). The *G*. *rostochienis*, *P*. *coffeae*, and *H*. *aoronymphium* sequences were extracted from unannotated genome data. Identical sequences were merged in a consensus sequence. EST, expressed sequence tag.
(XLSX)

S2 Table Overview of helminth VAL functional characterization. Collection of helminth VALs as discussed in this review with current nomenclature, structural information (including RCSB PDB reference [https://www.rcsb.org]) and information on lipid binding, interactions and function are given when available. PDB, Protein Data Bank; RCSB, Research Collaboratory for Structural Bioinformatics; VAL, venom allergen-like protein.
(XLSX)
