# Supplementary figures and images for: Secreted venom allergen-like proteins of helminths: Conserved modulators of host responses in animals and plants
Source: PLoS Pathog. 2018 Oct 18;14(10):e1007300. doi: 10.1371/journal.ppat.1007300 (PMC6193718; doi:10.1371/journal.ppat.1007300)

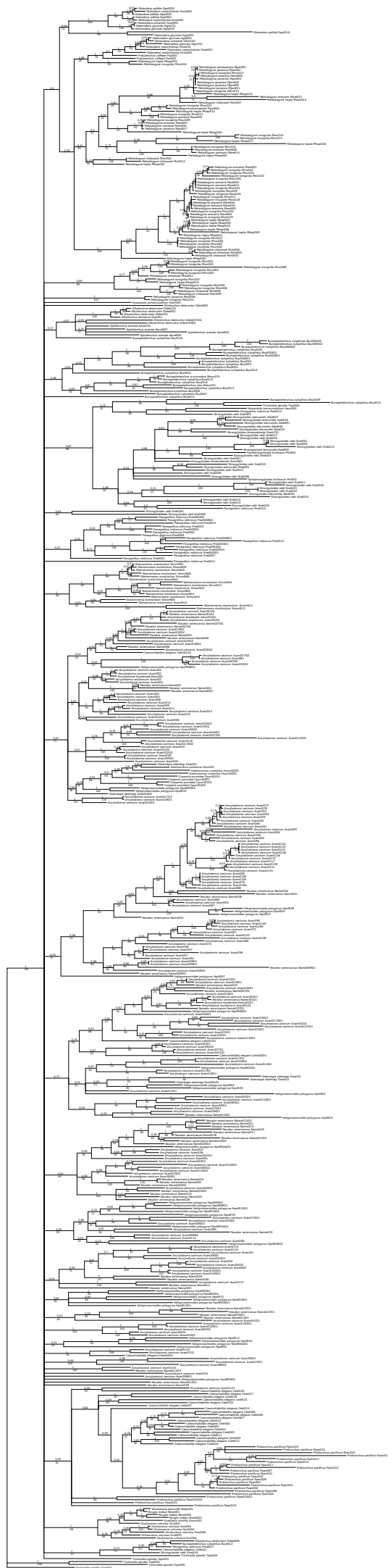

Supplement: S1 Fig — For multidomain proteins, each domain was included separately. Trichinella spiralis served as an outgroup. The alignment was created in BioEdit version 7.2.5 using ClustalW 1,4 and was further manually refined. The Bayesian tree was created using MrBayes version 3.2.6 and run for 10 million generations with 4 chains in 4 parallel runs using a mixed amino acid substitution model. Runs converged after a burnin of 2 million generations and used the WAG substitution model. Posterior probabilities are given above the branches. Displayed below the branches are the bootstrap percentages of a fast maximum likelihood tree run on the same dataset with RAxML version 8.2.10 using the WAG substitution model with 1000 bootstraps. All sequences that were used to constrict this tree are listed in S1 Table. VAL, venom allergen-like protein; WAG, Whelan and Goldman. (PDF) [file ppat.1007300.s001.pdf]
